# Short tandem repeat (STR) variation from 6 cities in Iraq based on 15 loci

**DOI:** 10.1186/s43141-023-00570-1

**Published:** 2023-12-05

**Authors:** Majeed A. Sabbah, Mohammed M. Al-Zubaidi, Thooalnoon Y. Al-janabi, Dhuha S. Namaa, Haider K. Al-rubai, Hala K. Ibrahem

**Affiliations:** Forensic DNA Research and Training Center, Al-Nahrain University, Jadriya, Baghdad, Iraq

**Keywords:** STR, Iraq population, Forensic DNA, Baghdad

## Abstract

**Background:**

One thousand sixty-one individuals were sampled from the cities of Anbar, Baghdad, Basra, Diyala, Najaf, and Wasit in Iraq and typed for 15 forensic STRs to explore the genetic structure of Iraq and develop a forensic DNA database. The total number of alleles that were identified was 203.

**Result:**

Analyses of molecular variance (AMOVA) were then conducted Baghdad provides a good representation of the rest of the country, while Anbar is the most genetically distinct. The average heterozygosities of these loci was 0.779, homozygosities was 0.221, polymorphism information content was 0.77, power of discrimination was 0.927, and power of exclusion was 0.563. At these loci, a matching genotype will occur, on average, in 1 in 8.152 × 1017 individuals. For paternity tests, the average paternity probability for a matching profile is 99.9997%.

**Conclusions:**

These loci are appropriate for use in forensic and paternity testing for this population. Iraq is similar to other countries in the Middle East, particularly Iran and Turkey, and is more similar to Europe than either Asia or Africa.

## Background

Short tandem repeats, also known as microsatellites, are repeated sequences of DNA, usually consisting of 2 to 6 bases. They are highly polymorphic and distributed throughout the genome. These sequences vary in the number of repeats, resulting in them being multiallelic [1]. Consequently, STRs prove very useful for forensic and paternity testing when multiple STR loci are employed [2]. The distribution of allele frequencies within the tested population enables the calculation of match probabilities. The objective of this study was to report allele frequencies for these STRs in the Iraqi population, facilitating their application in forensic and paternity investigations. The population of Iraq was estimated at 39 million in July 2017 [3]. Arabs constitute 75–80% of the population, while Kurds make up 15–20% [3, 4]. Other minorities, including Turkmen, Assyrian, Shabak, Yazidi, and more, are also present [3]. Samples were collected from various Iraqi cities: Diyala, Anbar, Wasit, Najaf, Baghdad, and Basra. Among these, Baghdad, Diyala, and Najaf are situated in the center of Iraq; Wasit is in the east; Basra is in the southeast; and Al-Anbar is in the west [4] and subjected to STR profiling.

## Methods

### Ethics committee approval and patient approval

The collection of samples used in the research (blood, buccal swab, saliva, and fingernails) was approved by the Center’s Scientific Research Ethics Committee before starting work. The volunteers signed a written consent to participate in this study.

### Sample collection

A total of 1061 unrelated individuals were sampled from six Iraqi cities: Diyala (*n* = 139), Anbar (*n* = 132), Wasit (*n* = 120), Najaf (*n* = 119), Baghdad (*n* = 354), and Basra (*n* = 198). The samples were collected as Buccal swabs from patients and laboratory workers at private laboratories. These samples were used to study the population genetic diversity in Iraq. The population genetics of Iraqis is important due to their ethnic diversity. Several studies have been conducted to analyze the genetic diversity of Iraqi populations, including studies on the distribution of Y chromosome haplotypes, 23-YSTR markers, and 15 STRs. These studies aimed to analyze the genetic structure of different cities in Iraq and to create a forensic DNA database for the country.

### DNA extraction

Samples were extracted using a PrepFiler Forensic DNA Extraction Kit (Applied Biosystems, Foster City, CA), and their DNA content was quantified with NanoDrop (Thomson, Wilmington, DE).

### PCR amplification

Fifteen autosomal STR markers (the 13 CODIS core loci and D19S433 and D2S1338) were genotyped along with the amelogenin locus on the X and Y chromosomes using the Applied Biosystems AmpFiSTR® Identifiler™ kit (3). Approximately 1ng of template DNA was amplified for each sample following the protocols described in the user’s manual (Applied Biosystems). The samples were amplified with an Applied Biosystems Veriti® PCR System (Applied Biosystems).

### DNA typing

Amplification products were diluted 1:15 in Hi-Di™ formamide and GS500-LIZ internal size standard (Applied Biosystems) and analyzed on the 16-capillary ABI Prism® 3100 Genetic Analyzer. On a 36-cm array, POP^TM^-4 (Applied Biosystems) was used for higher-resolution separations.

### Data collection

Data collection was performed with Data Collection v. 2.0 software (Applied Biosystems), and samples were analyzed with GeneMapper v. 3.2 software (Applied Biosystems) at the Forensic DNA Center for Research and Training of Al-Nahrain University.

### Statistical analyses

Allele frequencies for each locus, heterozygosities, homozygosities, polymorphic information anthropology, and content (PIC) measure the formativeness of a genetic marker, indicating how well it can distinguish between different alleles in a population. PIC = 1−∑ (pi^2) PIC ranges from 0 to 1, with higher values indicating greater allelic diversity and formativeness of the marker.

Matching probability (MP), powers of discrimination (PD) is a measure of how well a genetic marker can discriminate between individuals within a population.

Pi and pj are the frequencies of the *i*-th and *j*-th alleles at the locus, summed over all possible pairwise allele combinations.

PD also ranges from 0 to 1, with higher values indicating better discriminatory power. PD = ∑ (pi * pj).

Powers of exclusion (PE) measures the probability that two individuals randomly chosen from a population will have different genotypes at a specific locus.

PE = 1–P (same genotype)

*P* (same genotype) is the probability that two individuals chosen at random will have the same genotype at the locus.

PE can also range from 0 to 1, with higher values indicating a higher probability of distinguishing between individuals.

And typical paternity index (TPI) was calculated at each locus using Power Stats v1.2 [5]. The significance value for the HWE test was set at 𝛼 = 0.05 and *p* values were calculated using Monte Carlo methods with 100,000 permutations. The Holm-Bonferroni method [6] was used to account for multiple testing. Multidimensional scaling (MDS) was done using the “stats” package (R Core Team, 2016) to compare the Iraqi population to other Middle Eastern countries as well as countries from Europe, Asia, and Africa [7].

## Results

A total of 203 alleles were identified in this study. The distribution of these alleles across the 15 short tandem repeat (STR) loci is detailed in Table 1. Notably, notable occurrences include allele 8 of TPOX, which had the highest frequency at 51.0%, allele 12 of CSF1PO at 32.9%, allele 12 of D5S818 at 31.9%, allele 12 of D5S818 at 30.8%, allele 11 of CSF1PO at 30.7%, allele 11 of D16S539 at 30.6%, and allele 12 of D13S317 at 30.4%. It is noteworthy that the most diverse loci in terms of the number of distinct alleles were FGA with 23 alleles, D18S51 with 20 alleles, D2S1338 with 19 alleles, D19S433 with 17 alleles, and D21S11 with 17 alleles.

**Table 1:** Allele frequencies for STR loci D8S1179, D21S11, D7S820, CSF1PO, D3S1358, TH01, D13S317, D16S539, D2S1338, D19S433, vWA, TPOX, D18S51, D5S818, and FGA

Allele	D8S1179	D21S11	D7S820	CSF1PO	D3S1358	TH01	D13S317	D16S539	D2S1338	D19S433	vWA	TPOX	D18S51	D5S818	FGA
5	–			0.001		0.001									
6	–					0.280		0.001				0.003			
7	–		0.016	0.002	0.001	0.184		0.001				0.002	0.001		
8	0.009		0.165	0.004		0.127	0.149	0.040	0.001		0.001	0.510		0.008	
8.3	–					0.001									
9	0.006		0.109	0.026		0.248	0.071	0.171				0.127	0.002	0.057	
9.2										0.001					
9.3	–					0.136									
10	0.077		0.267	0.269		0.022	0.069	0.084	0.001	0.001	0.001	0.091	0.008	0.102	
10.2													0.001		
11	0.081		0.262	0.307	0.001	0.001	0.294	0.306		0.008	0.001	0.236	0.019	0.308	
12	0.104		0.159	0.329	0.001		0.304	0.241	0.001	0.089	0.001	0.029	0.132	0.319	
12.2										0.001					
13	0.267		0.020	0.052	0.003		0.076	0.136	0.001	0.234	0.004		0.174	0.194	
13.2										0.024					
14	0.193		0.002	0.008	0.055		0.033	0.020	0.001	0.251	0.077		0.173	0.011	
14.2										0.052		0.001			
15	0.202			0.001	0.267		0.002	0.001	0.001	0.139	0.107	0.001	0.135	0.001	0.001
15.2										0.094					
16	0.053			0.001	0.275	0.001	0.001		0.048	0.046	0.247		0.116		0.001
16.2										0.042			0.001		
17	0.007				0.253		0.001		0.193	0.011	0.286		0.097		
17.2										0.006					0.001
18	0.002				0.135				0.117	0.001	0.186	0.001	0.080		0.009
18.2										0.001			0.001		0.001
19	1.001				0.008			0.001	0.137		0.079		0.037		0.063
															0.001
20	–				0.001				0.141		0.012	0.001	0.012		0.095
															0.001
21	–								0.049				0.008		0.167
21.2	–														0.005
22	–								0.041				0.002		0.148
22.2	–														0.004
23	–								0.124				0.001		0.155
23.2	–														0.002
24	–								0.077				0.001		0.183
24.2	–														0.002
25	–							0.001	0.057		0.001				0.101
26	–	0.003						0.001	0.010						0.043
27	–	0.012							0.001						0.006
28	–	0.141							0.001						0.004
29	–	0.230													0.006
29.2		0.005													
30	–	0.235													
30.2	–	0.025													
31	–	0.045													
31.2	–	0.108													
32	–	0.005													0.001
32.2	–	0.135													
33	–	0.002													
33.2	–	0.047													
34	–	0.000													
34.2	–	0.005													
35	–	0.001													
36		0.000													

**Fig. 1:**
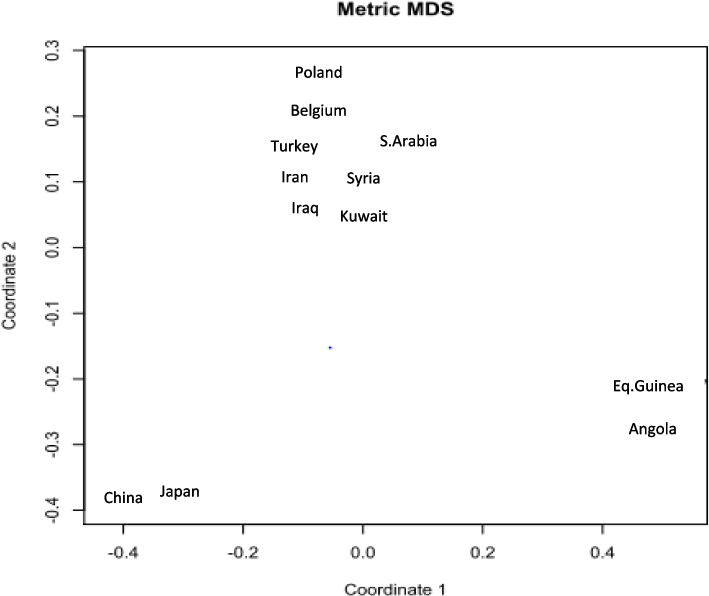
Multidimensional scaling plot of the genetic distances between Iraq, Turkey, Iran, Syria, Kuwait, Saudi Arabia, Poland, Belgium, China, Japan, Equatorial Guinea, and Angola

The city of Baghdad boasted the largest sample size, comprising 354 individuals. Interestingly, five alleles were exclusive to Baghdad and were not found in the other cities or neighboring countries. Three of these five alleles were absent from the other five Iraqi cities and all countries used for comparison, including Turkey, Iran [8], Syria [7], Kuwait [9], Saudi Arabia [10], Poland, Belgium [11], China [12], Japan [13], Equatorial Guinea, and Angola [14] (Fig. 1). Specifically, these alleles were allele 8 at vWA (found in Baghdad at 0.3%), allele 25 at vWA (found in Baghdad at 0.1%), and allele 16 at FGA (found in Baghdad at 0.3%). Baghdad also exhibited two other rare alleles: allele 11 at vWA (0.3% occurrence) and allele 15 at FGA (0.1% prevalence). Importantly, these alleles were absent in other Iraqi cities, Middle Eastern nations, and the European and Asian countries used for comparison [15]. However, they were present in the African countries used for comparison, such as Equatorial Guinea and Angola [16, 17]. Specifically, allele 11 at vWA was observed at 3.5% frequency in Equatorial Guinea and 0.4% in Angola, while allele 15 at FGA was found in Equatorial Guinea at 0.4%.

An additional rare allele, allele 22 at D13S317, was identified in the city of Wasit, with a frequency of 0.4%, but was not observed in any other Iraqi cities or countries in the comparison set [7].

To assess whether the observed genotype frequencies adhere to the expected frequencies, the population’s adherence to the Hardy-Weinberg equilibrium (HWE) was examined [18]. Table 2 displays the log-likelihood ratio *p* values for the HWE test [19, 20], which, after Holm-Bonferroni adjustment for multiple testing, were not deemed significant [21].

**Table 2:** Hardy-Weinberg Equilibrium *p*-values (for each five city) at each locus

Loci	Diyala	Anbar	Wasit	Najaf	Baghdad	South of Iraq
D8S.1179	0.97	0.94	0.69	0.07	0.15	0.56
D2.1S11	0.19	0.06	0.05	0.49	0.02	0.38
D7.S820	0.72	0.70	0.55	0.91	0.19	0.87
CS.F1PO	0.09	0.66	0.77	0.79	0.16	0.35
D.3S1358	0.04	0.95	0.10	0.37	0.08	0.07
TH.01	0.84	0.14	0.09	0.005	0.18	0.61
D13.S317	0.47	0.40	0.17	0.25	0.08	0.84
D16.S539	0.01	0.19	0.21	0.83	0.03	0.17
D2S.1338	0.03	0.64	0.98	0.37	0.001	0.07
D19.S433	0.45	0.40	0.77	0.54	0.68	0.64
vWA	0.22	0.53	0.93	0.65	0.11	0.39
TP.OX	0.31.0	0.12	0.07	0.78	0.01	0.52
D1.8S51	0.10	0.82	0.39	0.56	0.02	0.87
D.5S818	0.99	0.08	0.50	0.53	0.80	0.12
FG.A	0.26	0.82	0.48	0.04	0.01	0.15
Sample size	139	132	120	119	354	198

Details concerning each locus are provided in Table 3, encompassing heterozygosities (He), homozygosities (Ho), polymorphism information content (PIC), matching probabilities (MP), powers of discrimination (PD), powers of exclusion (PE), and the typical paternity index (TPI). All values fall within a range of 0.0 to 1.0, where 0.0 denotes the absence (or presence) of heterozygotes (or homozygotes) and 1.0 indicates full heterozygosity (or homozygosity) across the sampled individuals. The significance of these metrics varies with the genotype; high He, low Ho, substantial PIC, PD, and PE values characterize loci pertinent to forensic and paternity analyses. The average values for the 15 loci are He = 0.791, Ho = 0.209, PIC = 0.72, PD = 0.923, and PE = 0.587. Composite metrics, namely composite matching probability (CMP) and composite paternity index (CPI), are obtained by multiplying each locus’s MP and TPI, respectively.

**Table 3:** Forensic efficiency parameters for 15 STR loci (1061 samples) included matching probabilities, powers of discrimination, polymorphism information content, powers of exclusion, a typical paternity index, homozygosities, and heterozygosities

	MP*	PD*	PIC*	PE*	TPI*	Ho*	He*
D8S1179	0.052	0.948	0.80	0.619	2.64	0.189	0.811
D21S11	0.048	0.952	0.82	0.647	2.87	0.174	0.826
D7S820	0.072	0.928	0.76	0.561	2.27	0.221	0.779
CSF1PO	0.130	0.870	0.67	0.439	1.71	0.293	0.707
D3S1358	0.091	0.909	0.73	0.513	2.02	0.248	0.752
TH01	0.076	0.924	0.76	0.531	2.11	0.238	0.762
D13S317	0.790	0.921	0.75	0.570	2.32	0.216	0.784
D16S539	0.075	0.925	0.76	0.543	2.17	0.231	0.769
D2S1338	0.026	0.974	0.87	0.669	3.07	0.163	0.837
D19S433	0.045	0.955	0.82	0.629	2.72	0.184	0.816
vWA	0.068	0.932	0.77	0.520	2.05	0.244	0.456
TPOX	0.165	0.835	0.61	0.384	1.52	0.330	0.670
D18S51	0.029	0.971	0.86	0.646	2.85	0.175	0.825
D5S818	0.102	0.898	0.71	0.497	1.94	0.257	0.743
FGA	0.033	0.967	0.85	0.682	3.19	0.157	0.843
Sample size	1061	1061	1061	1061	1061	1061	1061

*MP matching probabilities, *PD* powers of discrimination, *PIC* polymorphism information content, *PE* powers of exclusion, *TPI* typical paternity index, *Ho* homozygosities, and *He* heterozygosities

Exploring STR analysis within Iraqi populations has been somewhat limited, and the studies that have been conducted employed distinct commercial STR kits, posing challenges for direct comparisons with the current study’s outcomes. Nevertheless, there is an overlap of the 15 STR markers between the aforementioned studies and neighboring countries, as outlined in Table 4.

**Table 4:** Comparison *p* values of HWE for STRs data for Arab-related populations

Locus	Iraq in this study	Jordan [22]	Turkey [23, 24]	Palestinian in Gazan [25]	Saudi Arabiae [10]	Iran [26]
	1061	95	350	125	190	274
D8S1179	0.80	0.774	0.814	0.83	0.81	0.81
D21S11	0.82	0.793	0.796	0.82	0.81	083
D7S820	0.76	0.694	0.775	0.77	0.75	0.78
CSF1PO	0.67	0.745	0.701	0.66	0.66	0.65
D3S1358	0.73	0.722	0.759	0.71	0.73	0.73
TH01	0.76	0.734	0.780	0.74	0.76	0.76
D13S317	0.75	0.654	0.750	0.73	0.72	0.75
D16S539	0.76	0.787	0.754	0.76	0.72	0.78
D2S1338	0.87	NS	0.877	N.S	0.84	0.87
D19S433	0.82	NS	0.881	N.S	0.85	0.82
vWA	0.77	0.802	0.794	0.78	0.78	0.78
TPOX	0.61	0.706	0.656	0.65	0.57	0.63
D18S51	0.86	0.878	0.864	0.86	0.82	0.87
D5S818	0.71	0.672	0.703	0.72	0.71	0.72
FGA	0.85	0.893	0.849	0.86	0.85	0.84

*NS* not significant

(−): not typed loci

Among these 15 common STR markers, D2S1338 demonstrated the highest Polymorphic Information Content (PIC) value within this study, while FGA exhibited the highest PIC value among Jordanians, succeeded by D19S433 in Turkey, D18S51 and FGA in Palestinians [25], and D19S433 and FGA in Saudi Arabia [27]. Conversely, the TPOX locus presented the lowest PIC value in this study, and within Jordanians [22], D13S317 displayed the lowest PIC value, followed by TPOX in Turkey [23], Palestinians, Saudi Arabia [27], and Iran [26].

## Discussion

In forensic investigations, CMP is frequently expressed as the likelihood that one individual in a certain population subset possesses a genotype matching the composite value. With the global population approximately at 7.5 billion, an average MP of 0.173 or lower across all 15 loci is necessary for the CMP to be interpreted as 1 in 7.5 billion or greater. The dataset's mean MP across 15 loci is 0.073. In paternity reports, the probability of paternity is determined by CPI/(CPI + 1), where a CPI of 100 or higher indicates a probability of 99.0% or higher. For the average TPI to reach 1.43 for 15 loci, the average TPI over 1.5 locations was 2.36 [20].

The findings of this investigation highlight the utility of these 15 autosomal STR loci as valuable markers for forensic and paternity testing within the Iraqi population. Within certain loci, the presence of a wide range of repeated sequences suggests possible population admixture dynamics. To elucidate the origins of the less common alleles discovered in this study, it may be beneficial to conduct further allele frequency analyses within specific ethnic groups in Iraq.

Lastly, the comparative evaluation of forensic genetic efficiency parameters across diverse populations, including the Middle Eastern region, is vital for comprehending genetic diversity and potential intermingling among these populations. To achieve a more accurate comparison, it is strongly recommended to conduct comparative studies employing the same STR kit across these populations, considering that different STR kits have been used previously to define forensic efficiency parameters.

### Consistency and variation

Looking at the values within each population, you can observe that some alleles have consistent frequencies across populations, while others vary more. This variation could be due to a variety of factors, including historical migrations, genetic drift, and natural selection.

### Genetic diversity

The range of allele frequencies across the different populations indicates the genetic diversity present in these regions. Higher diversity might be indicative of a more mixed or heterogeneous population.

The findings from this study highlight the effectiveness of the 15 autosomal STR loci as markers for forensics and paternity testing within the Iraqi population. It is worth noting that certain loci exhibit a wide range of repeats, which may be indicative of population admixture. To gain insight into the origins of the less common alleles identified in this study, further investigations should focus on analyzing allele frequencies within specific ethnic groups in Iraq.

A comprehensive assessment of forensic genetic efficiency parameters is crucial for understanding genetic diversity and admixture among different populations, including those in the Middle East. To ensure a robust comparison, it is strongly recommended that comparative studies utilize the same STR kit across these populations. Previous studies have employed disparate STR kits, which has affected the description of forensic efficiency parameters.

## Conclusion

The study discussed in the given text highlights the effectiveness of 15 autosomal STR loci as markers for forensics and paternity testing within the Iraqi population. The CMP represents the likelihood that an individual in a specific population subset possesses a genotype matching a composite value. With an average Match Probability (MP) of 0.073 across all 15 loci in a global population of 7.5 billion, the CMP can be interpreted as 1 in 7.5 billion or greater The presence of a wide range of repeated sequences within certain loci suggests possible population admixture dynamics. To understand the origins of less common alleles, further allele frequency analyses within specific ethnic groups in Iraq are recommended. Conducting comparative evaluations of forensic genetic efficiency parameters across diverse populations, including those in the Middle East, is essential for comprehending genetic diversity and potential intermingling among these populations. Using the same STR kit for these studies is crucial for accurate comparisons. The study also highlights that some alleles have consistent frequencies across populations, while others exhibit more variation, which could be attributed to factors such as historical migrations, genetic drift, and natural selection.

## Data Availability

All data analyzed during this study are included in this article.

## Abbreviations

STR: Short tandem repeat
AMOVA: Analysis of molecular variance
CODIS: Combined DNA index system
PIC: Polymorphic Information Content
MP: Matching probability
PD: Power of discrimination
PE: Probability of exclusion
PI: Paternity index
HWE: Hardy-Weinberg Equilibrium
